# Enhanced functional connectivity between the default mode network and executive control network during flow states may facilitate creativity and emotional regulation, and may improve health outcomes

**DOI:** 10.3389/fnbeh.2025.1690499

**Published:** 2026-01-09

**Authors:** Kelly Barnett, Fabian Vasiu

**Affiliations:** 1 Balance Medical Center, Vancouver, BC, Canada; 2 On Treks, Cluj-Napoca, Romania

**Keywords:** creativity, default mode network, emotional regulation, executive control network, flow state, functional connectivity

## Abstract

**Introduction:**

Flow is characterized by complete immersion and optimal engagement in a task, striking a balance between challenge and skill. Recent neuroimaging studies suggest that flow involves dynamic interactions among large-scale brain networks, particularly the default mode network (DMN) and the executive control network (ECN). This review aims to synthesize current findings on how flow-related DMN–ECN connectivity supports creativity and emotional regulation (ER).

**Methodology:**

Following PRISMA guidelines, we searched PubMed, PsycINFO, and Google Scholar for peer-reviewed neuroimaging studies that experimentally induced or measured flow states. Inclusion criteria encompassed task-based and resting-state fMRI, PET, or EEG designs focusing on DMN, ECN, or related networks (e.g., salience, reward), and studies explicitly reporting on creativity or ER outcomes. We extracted data on sample characteristics, flow induction methods, neuroimaging modalities, and main findings regarding DMN/ECN activation and connectivity. Risk of bias was assessed in the domains of selection, performance, detection, attrition, and reporting.

**Results:**

Nine studies met the inclusion criteria. Across diverse tasks—ranging from video games to jazz improvisation—flow was consistently associated with (1) down-regulation of core DMN regions (e.g., medial prefrontal cortex, posterior cingulate cortex) linked to diminished self-referential thought, (2) increased activity in lateral prefrontal and parietal areas underpinning attentional control, and (3) functional connectivity between networks often considered anti-correlated (e.g., DMN and ECN). This integrated network state appears to facilitate simultaneous idea generation (DMN) and goal-directed processing (ECN), supporting creativity. Additionally, reduced amygdala activity and insula–reward network coupling during flow suggest potential benefits for emotional regulation, allowing high focus and low anxiety.

**Conclusion:**

Flow emerges as a unique neurocognitive phenomenon marked by selective DMN suppression and enhanced ECN engagement. Such network reconfiguration fosters creativity through DMN–ECN synergy while providing emotional stability *via* reduced self-monitoring and negative affect. Although these findings are promising, further research should employ larger, more diverse samples, incorporate causal and longitudinal designs, and explicitly measure ER outcomes. Elucidating the neurochemical underpinnings of flow (e.g., dopamine release) and individual differences in “flow-proneness” remains an important future direction.

## 1 Introduction

### 1.1 Background

The concept of the flow state was first introduced by psychologist Mihaly Csikszentmihalyi in the 1970s to describe a mental state in which an individual is fully immersed in an activity, experiencing a sense of energized focus, full involvement, and enjoyment in the process (Csikszentmihalyi et al., 2014). During flow, people often lose awareness of time and self-consciousness, becoming deeply engaged in the task at hand. This optimal experience arises when there is a balance between the challenge of the activity and the individual’s skill level, clear goals, and immediate feedback. Key characteristics of flow include intense concentration, merging of action and awareness, a sense of control, and intrinsic reward from the activity itself.

The neural correlates of the flow state have been linked to several brain systems and networks. One such network is the reward system. One study investigated flow states in the context of balancing task difficulty and individual ability in a naturalistic video game setting (Huskey et al., 2018). Using fMRI to assess brain activity, the researchers identified that flow occurs when task demands and individual abilities are balanced. This balance corresponds to heightened functional connectivity between the executive control network (ECN)—centered in regions like the dorsolateral prefrontal cortex (DLPFC)—and the reward network, which includes the nucleus accumbens. The findings support the synchronization theory of flow (STF), which posits that flow emerges from the efficient integration of cognitive control and reward networks, driving the rewarding and immersive qualities of this state.

Attentional networks also appear to play a significant role in flow. In one study using two simple video games, Tetris and Pong, as experimental tasks to manipulate difficulty levels and measure flow experiences, participants played the games under four conditions: easy, optimal, hard, and self-selected (autonomy condition), with the optimal condition designed to match task difficulty with individual skill levels (de Sampaio Barros et al., 2018). Key findings revealed that the optimal difficulty condition elicited the highest self-reported flow feelings, accompanied by increased oxygenated hemoglobin concentrations in the frontoparietal attention network, specifically the right lateral frontal cortex and the right inferior parietal lobe. These results suggest frontoparietal regions play an important role in sustaining attention and supporting flow experiences. Conversely, the easy and hard conditions, which lacked the optimal challenge-skill balance, resulted in reduced flow feelings and lower activation of the frontoparietal network, suggesting that flow emerges when tasks are neither underwhelming nor overwhelmingly difficult.

The default mode network (DMN) has also been implicated in flow. A study linking flow with decreased neural activity included 27 participants who performed arithmetic calculations under three conditions: boredom, flow, and overload (Ulrich et al., 2014). Task difficulty in the flow condition was dynamically adjusted to match each participant’s skill level. Using perfusion imaging to measure regional cerebral blood flow (rCBF), researchers observed significant decreases in activity in the medial prefrontal cortex (mPFC) and the amygdala during the flow condition compared to boredom and overload. The study identified a distinct “U-shaped” pattern of rCBF in DMN regions, including the mPFC, angular gyri and parahippocampal cortex. The reduced mPFC activity was associated with diminished self-referential processing, a key characteristic of altered states of consciousness. Simultaneously, the decrease in amygdala activity correlated with lower arousal levels, perhaps contributing to the positive emotional experiences reported during flow. These neural changes were specifically tied to the flow condition, as they were absent during both boredom and overload, supporting the idea that transient reductions in prefrontal and emotional network activity underpin altered states of consciousness.

In another study, the DMN’s activity was explored under conditions of boredom, sustained attention, and rest (Danckert and Merrifield, 2018). Boredom, marked by disengagement from the task at hand, demonstrated DMN activation, particularly in the posterior cingulate cortex (PCC) and mPFC. These areas were consistently active in the boredom and resting-state conditions, aligning with their role in self-referential and internally focused thought. The anterior insula exhibited anticorrelated activity during boredom and sustained attention tasks, suggesting a failure to engage ECNs when confronted with mundane or repetitive stimuli. This pattern was absent in resting-state scans, where no external engagement was required, emphasizing the role of the DMN in differentiating between engaged and disengaged states. These findings underscore the DMN’s dynamic role in mediating cognitive states, from disengagement to optimal engagement, thereby contributing to our understanding of its function in altered states of consciousness like flow.

Finally, beyond reward and attentional accounts, flow has also been linked to executive/frontoparietal control systems (ECN) that sustain goal-directed focus. Across flow-induction paradigms, flow is typically associated with reduced activity in core DMN hubs (e.g., mPFC/PCC) alongside engagement of task-positive control regions (Ulrich et al., 2014; Ulrich et al., 2016), while work on creativity shows that default–executive cooperation can emerge when internally generated ideas must be shaped by task constraints (Beaty et al., 2016; Beaty et al., 2018).

According to a meta-analysis conducted by Alameda et al. (2022), our understanding of the neuronal activity during flow remains inconsistent—some studies suggest suppression of prefrontal activity in line with the transient hypofrontality hypothesis (THH), while others propose enhanced synchronization between attentional and reward-related networks, as suggested by the STF. The cerebellum has also been implicated, particularly in the internal model of flow, which posits that the flow state arises from efficient motor and cognitive execution governed by cerebellar models rather than explicit prefrontal control. EEG studies have identified changes in brain oscillations, particularly increased alpha and theta power in frontocentral regions, potentially reflecting heightened focus and cognitive efficiency. Methodological limitations observed across studies include small sample sizes, difficulty in reliably inducing flow in controlled settings, and reliance on indirect flow measures.

### 1.2 Flow and creativity

Flow may positively impact creativity. For example, a study of visual art tasks found that individuals who experienced more flow during the task also reported higher levels of creative thinking about their work (Cseh et al., 2015). More specifically, flow was highly correlated with participants’ self-rated creativity, indicating that when people enter a flow state, they subjectively feel more creative. Notably, the same study found flow was not directly tied to external judges’ ratings of the artwork’s creativity, suggesting flow’s main benefits may lie in motivation and engagement rather than immediate creative output.

The flow state has been linked to transient hypofrontality. When in flow, individuals often report a loss of self-consciousness and an altered sense of time, which aligns with reduced activity in frontal self-monitoring circuits. Arne Dietrich’s neurocognitive model of flow explicitly proposed that **a** prerequisite to the experience of flow is a state of transient hypofrontality, meaning the prefrontal cortex temporarily quiets down during deep flow states (Dietrich, 2004). This hypothesis has been supported by a recent neuroimaging study that recorded EEG brain activity in jazz musicians as they improvised music and rated their level of flow; the researchers found that high-flow states were accompanied by decreased frontal lobe activity. More specifically, high-flow (relative to low-flow) improvisations were associated with transient hypofrontality, confirming that frontal executive regions were less active during intense flow (Rosen et al., 2024). This reduction in frontal activity during flow is thought to suppress self-critical thoughts and deliberation, thereby allowing the person to fully immerse in the task at hand.

One possible explanatory mechanism for the findings above is the following: when someone enters a flow state, their brain may enter a mode of reduced frontal oversight (hypofrontality), which in turn frees up implicit, automatic processes to generate creative ideas or actions.

### 1.3 Flow and emotional regulation

Entering a flow state is often associated with a reduction in negative emotions and an increase in positive emotions. Rankin et al. (2019) found that individuals who experienced more flow during a stressful waiting period (e.g., law graduates awaiting bar exam results) reported significantly less worry, fewer negative emotions, and more positive emotions. In other words, engaging in a deeply absorbing activity served as an effective emotion-regulation strategy, distracting people from anxiety and improving their emotional state. Similarly, in a quasi-experimental study, performing musicians who underwent training to develop flow skills showed increased flow levels and decreased music performance anxiety, with an inverse relationship observed between flow and anxiety (Moral-Bofill et al., 2022). This inverse flow–anxiety link aligns with Csikszentmihalyi’s flow theory, which posits that when challenges and skills are balanced, people enter flow and anxiety is minimized.

Just as flow can shape one’s emotional state, a person’s emotion regulation skills can influence their ability to reach and maintain flow. Research indicates that individuals with stronger emotional regulation capacities—often measured as trait emotional intelligence—tend to experience flow more frequently. For example, Rakei et al. (2022) observed in a sample of musicians that trait emotional intelligence was positively correlated with dispositional flow proneness, whereas trait anxiety was negatively correlated with flow proneness. In fact, musicians with better emotional regulation (high emotional intelligence) were more likely to get into flow during performance, suggesting that managing one’s emotions (and keeping anxiety in check) is conducive to entering flow states.

### 1.4 Rationale for the review

Building on evidence that flow reduces self-referential DMN activity while engaging executive control processes, a key open question is whether flow also involves functional integration between DMN and ECN. We noticed a lack of comprehensive synthesis that specifically examines the functional connectivity between the DMN and ECN during flow states and its implications for creativity and ER. This review aims to address this gap by systematically analyzing neuroimaging studies that investigate the neural dynamics of flow, creativity, and ER, thereby providing an enhanced understanding of how these processes are interlinked at the neural level.

### 1.5 Objectives

The primary objective of this study is to systematically review and synthesize evidence on the functional connectivity between the DMN and the ECN during flow states and how this connectivity facilitates creativity and ER.

The secondary objective of the same is to examine the relationship between flow states and creative performance, identifying neural correlates that support enhanced creativity. We seek to explore how flow states influence emotional regulation mechanisms at the neural level, contributing to improved psychological wellbeing.

## 2 Methods

### 2.1 Search protocol

This systematic review was conducted following the Preferred Reporting Items for Systematic Reviews and Meta-Analyses (PRISMA) guidelines (Vrabel, 2015).

Included:

- Peer-reviewed studies
- Neuroimaging studies utilizing techniques such as fMRI, PET, or EEG.
- Study designs: Randomized controlled trials (RCTs), quasi-experimental studies, cohort studies, and cross-sectional studies.

Excluded:

- Reviews, meta-analyses, qualitative studies, conference abstracts without full data, case reports, editorials, studies in languages other than English, and non-human studies.

### 2.2 Search strategy

The literature search was conducted across multiple electronic databases, namely PubMed, PsycINFO, and Google Scholar. The following search queries were entered using Boolean operators (AND, OR) to combine the keywords:

- *Flow States:* (“flow state” OR “flow experience” OR “psychological flow”)
- *Default Mode Network (DMN):* (“default mode network” OR DMN)
- *Executive Control Network (ECN):* (“executive control network” OR ECN)
- *Functional Connectivity:* (“functional connectivity” OR “brain connectivity”)
- *Creativity and Emotional Regulation:* (creativity OR “emotional regulation”).

To maximize yield, we expanded this query with additional synonyms and related terms for each key concept. For “flow,” we included MeSH terms and alternatives (“optimal experience” and “autotelic experience”). For the “default mode network,” we used words referring to specific components “default network,” “medial prefrontal cortex (mPFC),” “posterior cingulate cortex (PCC).” Similarly, “executive control network” was broadened [“frontoparietal network,” “dorsolateral prefrontal cortex (DLPFC),” “anterior cingulate cortex (ACC)”]. We also included terms for “functional connectivity” (“connectivity patterns,” “network connectivity”) and expanded creativity (“divergent thinking,” “innovation”) and “emotion regulation” (“affect regulation,” “emotional control”). Finally, we explicitly included neuroimaging modality keywords (“fMRI,” “functional MRI,” “PET,” “EEG,” “electroencephalography”) to filter for studies using these techniques.

### 2.3 Data analysis

For each included study, we extracted key data: sample characteristics (including any relevant traits like expertise level), experimental task and flow induction method, neuroimaging modality and analysis techniques (with emphasis on connectivity analyses between DMN and ECN or related regions), main findings related to flow (especially changes in DMN/ECN activity or connectivity), any creativity or emotion-related outcomes, and authors’ noted limitations. We also recorded whether the study assessed or reported subjective flow (to confirm that neural findings correspond to actual flow experiences). Where available, we noted if the study examined correlations between neural measures and creativity or emotion measures.

Finally, we documented any potential biases (e.g., sample selection issues, blinding) to inform the risk of bias assessment. Given the variety of designs (mostly within-subject experiments and cross-sectional analyses), we evaluated risk of bias in the domains of the following:

- Selection Bias: Representativeness of the sample and whether any non-random group allocations could bias results. For instance, were the groups comparable (in studies comparing expert vs. novice)? Were participants appropriately recruited and, if applicable, randomly assigned to conditions?
- Performance Bias: Blinding of participants and personnel to the condition, and consistency of experimental conditions. Complete blinding in flow studies is often not feasible (participants know the task difficulty), but we noted that instructions or task order might influence performance (e.g., fixed order of easy/hard tasks could bias who experiences flow when).
- Detection Bias: Blinding of outcome assessment and objectivity of measures. Here, we considered whether brain image analysis was automated/unbiased and if subjective outcomes (flow self-reports, creative performance ratings) were obtained in a blinded manner.
- Attrition Bias: Incomplete outcome data, such as participants dropping out or data loss (e.g., excessive motion in fMRI) and how that was handled.
- Reporting Bias: Selective reporting of outcomes. We checked if the study’s results section matched its methods/hypotheses (to detect any undeclared selective omission of non-significant findings, for example). Pre-registration (if mentioned) was also considered.

Each study was rated as having low, unclear (some concerns), or high risk of bias in each domain.

## 3 Results

### 3.1 Overview

**Figure 1:**
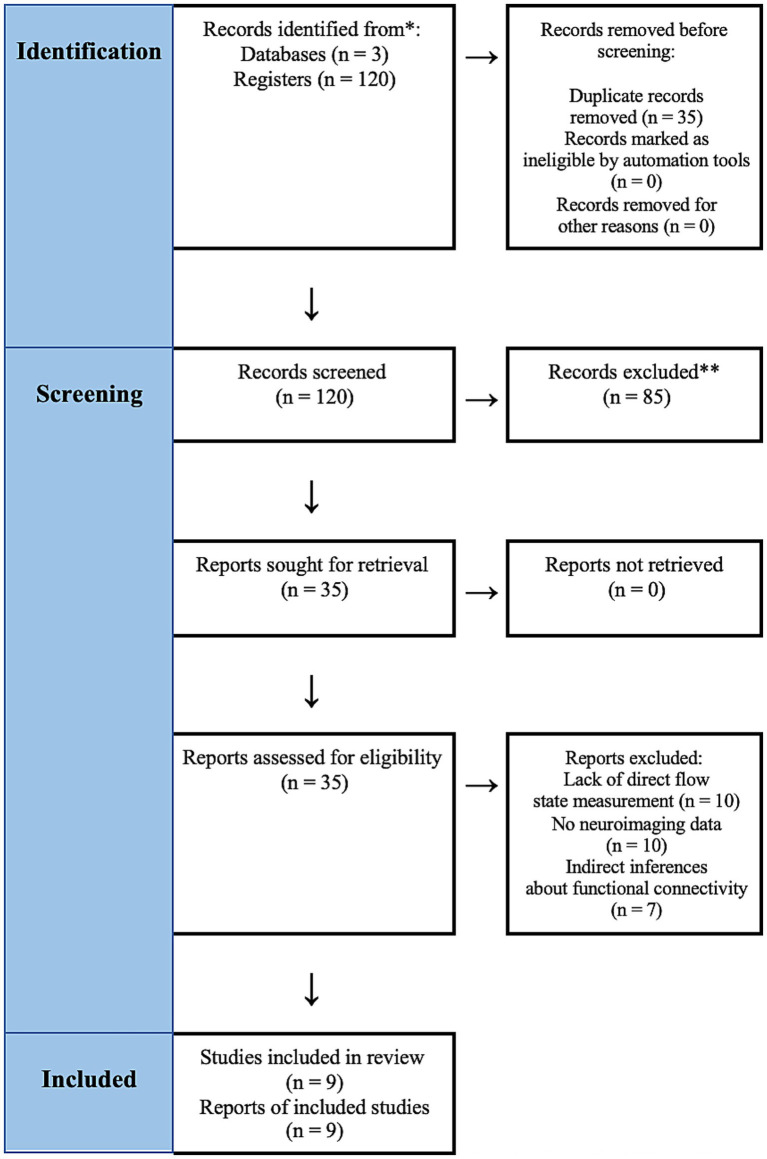
Study selection process. * indicates that records identified from databases and registers might include the same studies. ** indicates that exclusions occurred at the screening stage.

The initial database search yielded a total of 120 records (after removing duplicates). We screened titles and abstracts for relevance to flow, creativity/emotion, and neuroimaging. At this stage, 85 records were excluded for not meeting criteria (e.g., clearly not about flow or not a neuroimaging study, or being a review/meta-analysis). A total of 35 articles remained for full-text evaluation. After a thorough review of full texts, an additional 27 studies were excluded. The most common reasons were as follows: the study did not actually induce or measure a flow state (15 studies), did not involve an analysis of DMN or ECN specifically (five studies), or did not report functional connectivity results (four studies). We also excluded a few studies due to being non-English (two studies) or lacking peer review (one preprint). Ultimately, nine studies met all inclusion criteria and were included in the qualitative synthesis (Figure 1).

All eight studies that met our inclusion criteria were published between 2014 and 2024, reflecting the growing yet still emerging nature of neuroimaging research on flow states. The studies represent a mix of experimental paradigms used to induce flow, from video game scenarios and mathematical tasks to musical improvisation. Table 1 provides a summary of each included study and its primary focus.

**Table 1:** Summary of studies.

Study	Description
Ulrich et al. (2014)	Used perfusion fMRI (arterial spin labeling) to compare brain activation during an arithmetic task under “flow” (difficulty matched to skill) vs. “boredom” (easy) and “overload” (too hard) conditions. Focused on identifying brain regions whose activity decreases or increases during flow.
Ulrich et al. (2016)	Replicated and extended Ulrich 2014 using BOLD fMRI with shorter task blocks (30 s) in a within-subject design. Examined whether flow-related brain activation patterns (especially in DMN and ECN regions) could be detected with typical fMRI timing and confirmed physiological markers (electrodermal activity) of flow.
Ulrich et al. (2022a)	Employed a combined task-based activation and connectivity fMRI analysis to investigate the right anterior insula’s role as a salience network hub during flow. Flow was induced *via* a dynamic task (similar to challenge-skill balancing). The study specifically looked at changes in connectivity between the right insula and nodes of the ECN (DLPFC) and the DMN (mPFC) during flow vs. boredom/overload.
Huskey et al. (2018)	Tested the “synchronization theory of flow” using a naturalistic video game. Manipulated the balance between task difficulty and player skill in an open-source game and measured brain activity with fMRI. Focused on whether the flow condition (balanced difficulty) produces increased functional connectivity between cognitive control (ECN) regions and the reward network, compared to imbalanced conditions, which were hypothesized to activate the DMN (associated with disengagement).
de Sampaio Barros et al. (2018)	Investigated attentional resource mobilization during flow using near-infrared spectroscopy (NIRS) and psychophysiology. Participants played simple video games (Tetris, Pong) at easy, optimal (flow), hard, and self-chosen difficulty levels. Measured self-reported flow, attentional lapses, autonomic activity, and cortical oxygenation in frontoparietal regions to see how optimal challenge (flow) affects frontal and parietal activation and attention.
Beaty et al. (2016)	Examined whole-brain functional connectivity during a divergent thinking task to test if creativity is supported by cooperation between default mode and executive networks. Using fMRI, they compared a creative idea generation condition to a control condition, analyzing connectivity between key regions. Focus was on whether higher creative performance is associated with increased DMN–ECN coupling.
Beaty et al. (2018)	Used resting-state fMRI in a large sample to identify brain network predictors of trait creative ability. Although not an induced flow study, it was included for relevance to creativity. Found that individuals with higher creativity showed a whole-brain network linking default, salience, and executive regions working in concert.
Rosen et al. (2024)	EEG in 32 jazz guitarists (varying expertise) as they engaged in musical improvisation. Aimed to isolate brain oscillation patterns unique to the creative flow state. Compared experts (who more readily achieve flow) to less experienced players. Focused on neural signatures of “letting go” of executive control (transient hypofrontality) and the engagement of specialized creative networks when players experienced high-flow vs. low-flow moments.
Ulrich et al. (2022b)	Replication study of the established mental-arithmetic flow paradigm (boredom–flow–overload) using BOLD fMRI in a fresh sample (*N* = 41 healthy male participants). The study quantified replication evidence using the replication Bayes factor, reporting strong replication evidence for electrodermal activation and decisive replication evidence for both canonical neural “flow effects.” Inverted U-shaped activation was observed in regions including dorsolateral prefrontal cortex, anterior insula, and parietal cortex, while U-shaped activation was predominant in regions including medial prefrontal cortex, ventral striatum, amygdala, and cingulate cortex.

As shown in Table 1, all included studies explored flow in the context of a cognitively demanding activity (e.g., problem solving, gaming, creative performance) and measured brain function. Six studies used fMRI during active tasks, one used fMRI at rest (correlating intrinsic connectivity with creativity), one used NIRS (which measures cortical blood oxygenation) during tasks, and one used EEG during a real-world creative task (music improvisation). The sample sizes ranged from small neuroimaging samples of highly skilled individuals (e.g., 6–32 participants) to moderate samples (~41 participants). Notably, several fMRI studies (Ulrich et al., 2016; Ulrich et al., 2022a; Ulrich et al., 2022b) included only male participants, while others had mixed genders. The tasks to induce flow varied: three studies (Ulrich et al., 2016; Ulrich et al., 2014; Ulrich et al., 2022b) used a mental arithmetic task with adaptive difficulty, two used video games [a first-person shooter in Huskey et al. (2018); puzzle games in de Sampaio Barros et al. (2018)], one study (Rosen et al., 2024) involved musical improvisation, and Beaty et al. (2016) employed a creative brainstorming task. Despite different contexts, a common aim was to contrast a high-flow condition (or high-flow individuals) against low-flow comparisons (boredom, overload, novices, or control tasks) to identify neural differences.

All studies explicitly or implicitly examined the DMN and ECN. Some did so by measuring functional connectivity between nodes of these networks (e.g., DLPFC with posterior cingulate), while others inferred their involvement through activation patterns (e.g., decreases in mPFC/PCC activity as a sign of DMN deactivation during flow). Most studies also evaluated auxiliary networks or regions: for example, the salience network (right anterior insula) was a focus in Ulrich et al. (2022a), and reward circuitry (striatum) was examined in Huskey et al. (2018). Thus, while DMN and ECN are central to our review, the included studies consider flow within a broader network framework (DMN–ECN coupling, or DMN–salience–ECN interactions). Creativity outcomes were directly measured in two studies (Beaty et al., 2016; Beaty et al., 2018) and were also examined in an ecologically grounded creative-performance context in Rosen et al. (2024). The remaining flow-induction studies primarily focused on network activity/connectivity and affective or arousal-related proxies rather than behavioral creativity outcomes. Emotional aspects were typically addressed *via* neural proxies (e.g., amygdala activity for emotion, or self-reported stress/arousal).

Table 2 summarizes each study’s neuroimaging techniques, key findings (especially regarding DMN, ECN, and connectivity), and any limitations as noted by the authors or identified in our review.

**Table 2:** Neuroimaging technique, key findings, and limitations.

Study	Neuroimaging techniques used	Key findings	Limitations
Ulrich et al. (2014)	Perfusion fMRI (Arterial Spin Labeling) during mental arithmetic task. Within-subjects: boredom vs. flow vs. overload conditions (3-min blocks).	– Flow vs. others: Increased activity in left IFG (Brodmann 45) and left putamen; Decreased activity in mPFC and amygdala. – Interpretation: IFG activation = greater executive control; Putamen = reward/outcome anticipation. mPFC down = less self-referential thought, Amygdala down = lower arousal/negative emotion.	– Sample: *N* = 27 healthy students; limited diversity (possible gender imbalance not reported). – Task: Arithmetic may not generalize to all flow activities (e.g., creative arts). – Connectivity: Only regional activation measured; functional connectivity between regions was not analyzed (inferred DMN involvement only by deactivation pattern). – Flow measurement: Relied on task design and post-task ratings; real-time flow fluctuations are not captured.
Ulrich et al. (2016)	Task fMRI (BOLD) with short blocks (30 s) of boredom, flow, and overload in arithmetic task. fMRI contrasts + physiological measures (skin conductance).	– Flow activation: Significant increases in bilateral anterior insula, bilateral inferior frontal gyrus, basal ganglia (putamen/caudate), and midbrain during flow vs. non-flow. – Flow deactivation: Decreased BOLD in mPFC, PCC (DMN core nodes), and in medial temporal lobe, including amygdala. – Replicated U-shaped pattern: intermediate arousal (skin conductance) in flow, higher in overload, lower in boredom (supporting flow’s distinct physiological profile).	– Sample: *N* = 23, all male young adults, which limits generalizability to females or older populations. – Design: Task order possibly counterbalanced but not explicitly stated (if not, order effects could influence results). – Analysis: Still activation-based; no direct connectivity analysis (though authors discuss possible network interactions). – Flow validity: Short 30 s “flow” blocks may not fully capture sustained flow; however, subjective and EDA data confirmed participants did experience something akin to flow.
Ulrich et al. (2022a)	Task fMRI (BOLD) during a challenge-skill task, inducing flow, boredom, overload (block design). PPI connectivity analysis centered on right anterior insula (rAI).	– Connectivity (Flow vs. others): Increased functional coupling of rAI with left and right DLPFC (ECN) during flow. No flow-related coupling increase with mPFC or amygdala. – Decreased coupling of rAI with ventral striatum (ventral caudate/nucleus accumbens) during flow relative to boredom/overload. – Activation: rAI itself showed an “inverted U” activation (highest in flow, lower in boredom/overload); ventral striatum showed a U-shaped pattern (least active during flow). – Interpretation: The anterior insula orchestrates network switching: in flow, it strongly engages ECN (DLPFC) and disengages reward input (striatum) relative to non-flow. This supports the salience network driving focus in flow.	– Sample: *N* = 41, all males. Gender-specific effects are unknown. – Generality: Used a lab task; unclear if findings generalize to real-world flow (e.g., sports or arts). – Regions of interest: Focused on insula connectivity; may have missed other network interactions (e.g., direct DMN–ECN coupling) by not doing whole-brain connectivity analysis beyond rAI. – Subjective flow: Assessed (presumably) *via* questionnaires but not reported in detail in results—assuming flow manipulation was effective, but individual differences in experienced flow are not analyzed against connectivity.
Huskey et al. (2018)	Task fMRI (BOLD) using an open-source video game with varying difficulty. PPI connectivity analysis between cognitive control regions (e.g., DLPFC) and reward regions. Also measured self-reported intrinsic reward and flow.	– Balanced-difficulty (highest engagement) condition showed the highest self-reported intrinsic reward/engagement. – In PPI analyses using nucleus accumbens as the seed, connectivity differences were observed with frontal control-related regions when contrasting balanced vs. low/high difficulty. – Basal ganglia findings (including putamen) included an additional reaction-time component indexing engagement	– Multi-study design with a moderate fMRI sample, limiting precision/generalizability. – Naturalistic first-person game boosts ecological validity but may not generalize to other flow domains/non-gamers. – In parts of the paper, intrinsic reward/engagement is used as a proxy for flow, which is related but not identical. – PPI results are seed-dependent: effects were reported with nucleus accumbens as seed; when DLPFC was the seed, a significant DLPFC–accumbens link was not reported, so findings should not be framed as bidirectional control–reward coupling. – The added reaction-time task and large RT differences raise a dual-task confound, meaning basal ganglia findings may reflect control demands rather than reward per se. – Multiple analyses/contrasts increase interpretive complexity.
de Sampaio Barros et al. (2018)	NIRS (near-infrared spectroscopy) monitoring oxygenation in prefrontal and parietal regions + physiological measures (heart rate, HRV, breathing) during gameplay. Within-subjects: easy, optimal, hard, and self-selected difficulty conditions.	– Optimal (flow) vs. Easy/Hard: Greater self-reported flow and higher oxygenated hemoglobin concentration in right lateral PFC and right inferior parietal lobule (frontoparietal network) during optimal difficulty. Indicates increased activation of attentional control regions in flow. – Autonomy (self-chosen level): Did not increase flow beyond optimal but showed even greater physiological arousal (higher breathing rate, lower HRV) and high PFC/parietal activation. Suggests choice adds effort/stress without boosting flow feelings. – Attentional focus: Fewer task-unrelated thoughts (“mind-wandering”) were reported in the flow condition. – Conclusion: Flow is supported by mobilizing attentional resources in frontoparietal regions, enabling absorption. Autonomy can engage those resources but may introduce performance pressure, not necessarily increasing subjective flow.	– Technique: NIRS has limited depth (cortical surface only) and spatial resolution, so deeper DMN nodes (mPFC, PCC) were not directly measured—only lateral PFC and parietal regions. – Sample: *N* = 18, relatively small; all young adults. – Flow generalization: Used simple games (Pong, Tetris) —flow dynamics might differ in complex or long-term tasks. – No direct network measure: Although results imply frontoparietal (executive/attention) network engagement, no connectivity between regions or other networks was analyzed. – Autonomy condition: Order of conditions not clear—if autonomy was always last, effects could partly be due to fatigue or expectation.
Beaty et al. (2016)	Task fMRI (BOLD) during a creative divergent thinking task (“Alternate Uses” for common objects) vs. a control task (object characteristics listing). Analyzed whole-brain functional connectivity (using network-based analyses and seed-to-voxel) to identify network coupling related to creativity.	– Creative ideation vs. control: Increased functional connectivity between DMN and ECN regions during creative thinking. Notably, stronger coupling between medial prefrontal (DMN) and dorsolateral prefrontal (ECN) regions, and between inferior parietal (DMN) and inferior frontal (ECN) regions, was observed. This indicates cooperation between introspective and executive processes during creativity. – Resting-state follow-up: In a separate sample, resting connectivity analysis confirmed that the regions active in the creative task belong to distinct networks (default, executive, salience) and that individuals with higher creativity had more integrated networks at rest. – Divergent thinking ability: The efficiency of the identified creative brain network (including DMN and ECN nodes) correlated with participants’ divergent thinking scores. – Interpretation: The ability to generate creative ideas relies on concurrent engagement of spontaneous thought (DMN) and controlled processing (ECN)—a neural parallel to the flow experience of being freely imaginative yet task-focused.	– Flow not measured: Did not explicitly induce or measure “flow,” though participants were performing a creative task. Relevance to flow is inferred (creative immersion likely engages similar networks). – Sample: *n* = 163 across multiple analyses (including validation sample), which is robust. However, all were young adults; needs replication in other age groups. – Task design: Block design might allow mind-wandering even in control blocks; however, contrasts were carefully done. – Causality: Correlational fMRI; cannot determine if DMN–ECN connectivity causally improves creativity or is a byproduct of creative effort.
Beaty et al. (2018)	Resting-State fMRI connectivity + Creative ability assessment. Used connectome-based predictive modeling to link intrinsic network connectivity with divergent thinking scores across participants.	– Identified creative network: A whole-brain network spanning default, executive, and salience systems predicted creativity scores. Key hubs included medial prefrontal (DMN), lateral prefrontal (ECN), and insula (salience), among others. – Individuals with stronger connectivity within this tri-network circuitry had higher creative performance (measured by divergent thinking tasks scored for originality). – Provides additional evidence that default executive network coupling (with salience network mediation) is a trait marker of creativity. This complements state-based findings of network coupling during creative tasks.	– Not task-based: Did not involve a flow task, so direct conclusions about flow states cannot be drawn. Included here to support general link between network integration and creativity. – Sample: Adequate size (two samples, *n* ≈ 90 each). – Method: Predictive modeling is complex; results are robust, but the specific network connections contributing to the model are numerous and not all are easily interpretable (beyond noting DMN/ECN/Salience hubs). – Generality: Focus on divergent thinking; other kinds of creativity (artistic, interpersonal) are not directly tested.
Rosen et al. (2024)	EEG (64-channel) recorded during real-time jazz guitar improvisation. Compared neural oscillations in expert vs. less experienced musicians. Analyzed spectral power changes associated with self-rated flow levels.	– Expert musicians: Achieved flow more frequently and intensely than novices. Their EEG showed patterns of “optimal processing”: specifically, decreased beta power in frontal regions (less executive control interference) and increased alpha/theta synchronization in auditory and visual areas (heightened sensory immersion) during high-flow improvisation (based on press description) TECHEXPLORIST. COM. This reflects the idea of transient hypofrontality—experts in flow showed lower engagement of frontal control circuits, allowing creative processes to unfold more automatically. – Novice musicians: Required more cognitive control (likely higher frontal beta activity) and experienced fewer periods of true flow. Their performance was more effortful and accompanied by more self-monitoring (as inferred from less frontal deactivation). – DMN: The default mode regions were not directly measured (EEG limitation), but indirectly, the high-flow state in experts was linked with “letting go” of self-focused thinking—consistent with reduced DMN influence (as seen in fMRI studies). – Emotional state: High-flow performances were subjectively rated as more enjoyable and creatively satisfying.	– Ecological validity: High (real musical creation in lab), but at cost of experimental control. Each improvisation was unique; it was hard to ensure consistency of “challenge-skill balance” across individuals. – EEG source localization: Difficult to pinpoint deeper sources like DMN nodes; conclusions about DMN are indirect. – Flow measurement: Flow was likely assessed *via* self-report after each improvisation. Real-time changes had to be inferred rather than continuously rated, which could introduce some inaccuracy in aligning EEG data to flow states. – Group differences: Experts vs. non-experts differ in many ways (years of training, perhaps confidence, etc.), so some EEG differences may not solely be due to flow but expertise generally. However, within experts, clear neural changes from low- to high-flow improvisation strengthen the flow-specific interpretation.
Ulrich et al. (2022b)	Task-based BOLD fMRI (3 T; block design) during a mental arithmetic flow paradigm with three conditions (boredom, flow, overload) and adaptive difficulty; whole-brain task-activation GLM testing quadratic (inverted-U and U-shaped) contrasts across conditions; replication Bayes factor approach to quantify replication evidence for neural effects. Concurrent electrodermal activity (EDA) recording as a psychophysiological marker of the flow manipulation.	Reported strong replication evidence for the inverted-U-shaped EDA effect (highest sympathetic arousal in flow relative to boredom/overload). For neural activation, the study found decisive replication evidence for both canonical quadratic flow effects: (1) an inverted U-shaped activation pattern (greater activation during flow than boredom/overload) in regions including dorsolateral prefrontal cortex, anterior insula, and parietal cortex; and (2) a U-shaped activation pattern (lower activation during flow than boredom/overload) in regions including medial prefrontal cortex, ventral striatum, amygdala, and cingulate cortex (subgenual, middle, posterior).	Men-only sample (*N* = 41) limits generalizability; authors explicitly note the need for replication in women-only samples under hormonal control. Flow experience ratings could not be fully analyzed as a replication outcome due to an item implementation error (only 2/3 intended items were valid), reducing comparability on subjective flow indices. The replication Bayes factors were derived using a *p*-value-based upper-bound approximation to Bayes factors, which may overestimate absolute BF magnitudes (though the authors argue this should minimally affect the replication BF ratio). Finally, the paradigm uses mental arithmetic, so some inverted-U activations may partly reflect task demands rather than flow-specific processes.

As shown in Table 2, the studies suggest that the brain in flow is a better-optimized network, in which task-relevant systems are upregulated and task-irrelevant systems are downregulated or integrated in service of the task. A few limitations are common across studies: small sample sizes (typical in neuroimaging) and homogeneous samples (many studies used young, educated participants, some only males), which raise questions about generalizability. Additionally, several studies did not directly measure creativity outcomes or emotion regulation as behavioral variables; instead, they inferred them from neural proxies. Only two studies (Beaty et al., 2016; Beaty et al., 2018) explicitly linked brain connectivity to creativity metrics, and none directly measured emotion regulation ability. This suggests a gap for future research to connect these dots more explicitly (e.g., does inducing flow improve creative output or emotional resilience compared to a control state?). Another limitation is the issue of causality: almost all studies are correlational (with the exception of one that used brain stimulation in a related experiment, and one could consider the challenge-skill manipulations as causal factors for flow). We cannot be certain, for example, if DMN suppression is causing the flow state or is simply an effect of it (or both are being caused by something else, like dopamine levels). Nonetheless, the convergence of evidence across modalities (fMRI, EEG, NIRS) lends credibility to these neural patterns.

We assessed each study for potential biases that might affect the validity of its findings (Table 3).

**Table 3:** Risk of bias.

Study	Selection bias	Performance bias	Detection bias	Attrition bias	Reporting bias
Ulrich et al. (2014)	Low risk. Volunteer sample of students; within-subject design minimizes group differences. (All conditions experienced by all participants.)	Some concerns. Participants not blinded to task difficulty; possible expectation of “optimal” condition. Task order not specified (could influence engagement).	Low risk (objective perfusion MRI data; flow ratings used but likely collected uniformly).	Low (27 entered, 27 analyzed; no dropouts reported).	Low (All hypothesized regions reported, both increases and decreases).
Ulrich et al. (2016)	Some concerns. Sample of 23 male students only; limits external validity, though internally participants serve as their own controls.	Some concerns. Difficulty conditions are obvious to participants; short blocks might cause carry-over (counterbalancing unclear). Experimenters knew condition sequences.	Low risk (fMRI outcomes objective; flow verified *via* EDA and self-report collected similarly across conditions).	Low (all 23 analyzed; no mention of exclusions).	Low (Reported both activation and deactivation findings; analysis plan followed prior study, reducing selective reporting risk).
Ulrich et al. (2022a)	Some concerns. 41 male participants; results may not generalize to females. Otherwise, within-subject comparisons are sound.	Some concerns. Likely not blinded to condition; however, task was automated. Not sure if “flow” block could be anticipated by participants.	Low (connectivity analysis objective; investigators presumably blind during data processing. Self-report flow data not heavily featured).	Low (no dropouts reported; adequate data from all).	Low (Focus was on insula connectivity as pre-registered hypothesis; full results for that reported. Unreported analyses unlikely).
Huskey et al. (2018)	Low. Recruited gamers; all participants experienced all difficulty conditions. Groups not an issue (within-subject design).	Some concerns. Participants know when game is easy vs. hard. However, the engaging nature of the game might reduce demand characteristics.	Low (fMRI and psychophysiological data collected identically across conditions. Self-reports of challenge/skill likely obtained immediately, reducing bias).	Low (retention good; any data loss due to technical issues was minor).	Low (All relevant outcomes discussed, including cases where DMN increased in non-flow, supporting no selective omission).
de Sampaio Barros et al. (2018)	Low. Within-subject with counterbalanced game conditions (assumed; if not, could be an issue—but likely rotated orders). Sample is somewhat small (18) but acceptable.	Some concerns. Participants could identify easy vs. hard levels, which might affect effort. Also, if autonomy condition always lasts, there is an order bias. No blinding of experimenters to condition.	Some concerns. NIRS outcome objective, but flow feelings were self-reported (might bias toward thinking “optimal = flow”). However, probe questions were used to catch mind-wandering, which is a strength.	Low (no mention of dropouts; all provided data for each condition).	Low (Reported multiple measures—physiological, self-report, NIRS—even when some were non-significant; appears comprehensive).
Beaty et al. (2016)	Low. Large sample: all did both creative and control tasks in random order. Individual differences in creativity were accounted for in analysis.	Low. Task order was counterbalanced across subjects; participants were not aware of the specific hypothesis about network coupling.	Low (fMRI connectivity analysis is objective. Creativity outcomes (divergent thinking scores) were assessed outside scanner by independent judges).	Low (all participants included; data complete. Any with excessive motion likely removed per standard, not affecting bias).	Low (Well-reported: they published even null results for some analyses and did confirmatory analyses. No obvious selective reporting).
Beaty et al. (2018)	Low. Two independent samples are used for model training and testing, enhancing robustness. Participants were recruited without regard to creativity (range of scores achieved).	Low. Resting-state scan—no performance to bias. Participants did not know their connectivity was to be correlated with creativity, so no performance expectations.	Low (Functional connectivity at rest measured objectively; creativity testing was standardized and presumably blinded scoring).	Low (nearly all who were scanned provided usable data; those with high head motion were excluded per protocol, not likely systematic bias).	Low (Outcomes pre-specified: prediction of creativity. Paper reports both successful and unsuccessful predictions for comparison models; likely no selective omission).
Rosen et al. (2024)	Some concerns. Non-random two groups (experts vs. non-experts); differences could confound (e.g., age, music training aside from expertise). They did have both groups perform same task to mitigate that.	Some concerns. Impossible to blind musicians to their skill level or task; they knew they were improvising. Researchers obviously knew who an expert was. Performance setting was standardized, though.	Low (EEG data collection uniform. Flow ratings might be subjective, but experts vs. novices would not bias their own rating differently except as true reflection of experience. The experimenters scoring performances for creativity, if any, should ideally be blinded to expert/novice—unclear if done).	Low (32 entered, possibly all completed. If any EEG data were excluded, it was likely due to noise, unrelated to flow level).	Low (Main outcomes (frontal deactivation in experts, etc.) reported. Qualitative descriptions align with hypothesis. No indication that contrary data were hidden—novices’ data were reported as showing less of the effect, as expected).
Ulrich et al. (2022b)	Some concerns. Convenience sample of healthy male university students only (*N* = 41); limits generalizability (sex/age/culture). Within-subject design reduces between-group confounding.	Some concerns. Participants can infer condition difficulty (boredom/flow/overload), so expectation/demand effects are possible; however, two predefined block sequences were counterbalanced, reducing order effects.	Low risk. Primary outcomes are objective (BOLD fMRI + EDA). Subjective flow ratings were compromised by an item error and therefore not used for replication, which reduces risk of biased subjective outcome interpretation (but weakens manipulation-check depth).	Low risk. No dropouts/exclusions are indicated for the replication fMRI sample; analyses are reported for the full replication sample.	Low risk. Clearly framed as a confirmatory replication of pre-specified quadratic (U/inverted-U) effects with replication Bayes factor quantification; results presented for the primary effects and physiological marker.

Overall, the included studies were of moderate quality with some specific risks. All were lab-based experiments or observational studies; none were randomized controlled trials (except the experimental manipulations of task conditions, which were within-subject). Therefore, some RoB domains (like blinding) are inherently challenging.

### 3.2 Main findings

Despite using varied tasks and measures, the studies converged on a few consistent neural signatures of the flow state: (1) a relative deactivation or disengagement of core DMN regions (medial prefrontal, posterior cingulate) during flow, corresponding to reduced self-referential processing and distractions; (2) heightened activation of executive or task-positive regions (lateral prefrontal, intraparietal, and attention-related areas) to support focused engagement; and importantly (3) increased functional connectivity between networks that are usually at odds (such as the DMN and ECN), which may underlie the simultaneous demands of spontaneity and control in creative flow. Additionally, changes in emotional/reward centers (such as the amygdala and striatum) were observed, aligning with flow’s positive affect and intrinsic reward.

#### 3.2.1 Deactivation of default mode regions

Multiple studies found that being in flow correlates with suppression of brain areas associated with the DMN, particularly the mPFC. Ulrich et al. (2014) first showed this using perfusion MRI: during flow (vs boredom or overload), activity in the mPFC was significantly reduced. This was interpreted as diminished self-monitoring and reduced internal distraction, a hallmark of flow. They also noted a concomitant decrease in the amygdala during flow, which the authors linked to lower negative arousal, fitting the calm yet focused emotion in flow. In their follow-up BOLD-fMRI study, Ulrich et al. (2016) confirmed “relative activation decreases” in key DMN nodes (mPFC and posterior cingulate cortex, PCC) during flow blocks compared to non-flow conditions. Notably, the PCC is a major DMN hub involved in mind-wandering; its deactivation suggests fewer task-unrelated thoughts in flow.

Rosen et al. (2024) similarly reported decreased activity in certain DMN regions during moments of high creative flow in expert improvisers. In their EEG study, while specific brain regions are hard to localize, the press release noted that “decreased activity in certain regions of the default mode network” accompanied intense flow, implying that the DMN was less influential when artists were generating ideas effortlessly.

Together, these findings suggest that flow is characterized by a partial downregulation of the DMN, consistent with the subjective loss of self-consciousness and time distortion. By quieting the DMN, the brain may reduce self-evaluation and rumination, thus also facilitating a state of low anxiety and low stress.

#### 3.2.2 Engagement of executive/control networks

Complementing DMN deactivation, flow states showed increased activation in executive and attention networks. For example, Ulrich et al. (2014) found a relative increase in the left inferior frontal gyrus (IFG) during flow. The IFG (part of the lateral PFC) is implicated in attention and cognitive control; its activation aligns with the idea that flow involves intense concentration. They also observed increased activation of the putamen (a basal ganglia structure) in flow, possibly reflecting reward processing or fluent motor/cognitive sequencing. Later, Ulrich’s team showed that bilateral inferior frontal gyri and the anterior insula were significantly more active during flow than comparison states, along with parts of the basal ganglia and midbrain (Ulrich et al., 2016). The anterior insula is noteworthy because it is part of the salience network (SN), which toggles between DMN and ECN; its strong activation suggests heightened interoceptive and salience processing during flow, which may help maintain focus on the task at hand.

de Sampaio Barros et al. (2018) using NIRS found that the frontoparietal attention network was more engaged when participants played at optimal difficulty. Specifically, oxygenated hemoglobin was higher in the right lateral PFC and right inferior parietal lobe under the flow condition. This indicates greater recruitment of attentional resources during flow, supporting the idea that flow is an absorbed state requiring executive attention. In the context of creative flow, Rosen et al. (2024) reported that experienced musicians showed reduced activity in the executive control region of the brain (frontal cortex) only after they had acquired extensive skill—described as a “letting go” of overt control. This phenomenon, which is transient hypofrontality, might seem contradictory to other findings of PFC activation. However, it may reflect the difference between novices, who must exert conscious control to play, vs. experts, who can afford to suspend excessive conscious monitoring. In experts, flow was achieved by “releasing control”—the dorsolateral PFC (DLPFC) showed deactivation, presumably because control processes had become automatized. Interestingly, this deactivation in experts co-occurred with increased activation in sensory areas (visual and auditory regions) during flow, suggesting their brains devoted more resources to perceiving and integrating the music in real-time, rather than self-monitoring.

In summary, flow seems to involve a delicate balance of ECN involvement: engagement of control circuits to maintain focus and handle challenges, but in some cases, relative reduction in prefrontal executive oversight to allow fluid performance (especially in well-learned creative skills). This balance may depend on the level of skill and automaticity: novices ramp up PFC activity to meet a challenge, whereas experts in flow can “dial down” unnecessary PFC activity, preventing overthinking.

#### 3.2.3 DMN–ECN functional connectivity

A central question is whether flow involves increased integration between the DMN and ECN, as opposed to the usual anti-correlation seen at rest. Beaty et al. (2016) provided evidence in the context of creativity (not explicitly flow, but divergent thinking likely shares similarities): During creative idea generation, they found increased functional connectivity between regions of the default network and executive network, highlighting cooperation between these large-scale networks in supporting creative production. In other words, the brains of participants “in the creative zone” showed greater coupling of brain areas that typically belong to opposite networks—for example, coupling between the inferior prefrontal cortex (a control hub) and the posterior cingulate cortex/precuneus (a default mode hub). This suggests that creative thinking requires both spontaneous imagination from the DMN and the focused selection and evaluation from the ECN working in tandem. Beaty’s group then showed that individuals with high creativity have a capacity for simultaneous engagement of default, executive, and salience networks, implying that the ability to flexibly switch and integrate these networks is a hallmark of creative brain function.

Huskey et al. (2018) linked the balanced challenge–skill condition to higher subjective engagement and reported PPI effects using the nucleus accumbens as a seed, suggesting that striatal coupling with frontal control-related regions varies with engagement state. However, because significant DLPFC—accumbens coupling was not reported when DLPFC served as the seed, these results cannot be framed as bidirectional control–reward synchronization. In addition, the study’s secondary reaction-time task introduces the possibility that basal ganglia effects partly reflect dual-task control demands rather than reward processing per se.

Ulrich et al. (2022a) explicitly examined connectivity changes between networks using a flow paradigm. They focused on the right anterior insula (rAI), a hub of the salience network that often initiates switches between DMN and ECN. During flow (vs boredom/overload), the rAI showed increased coupling with the left and right DLPFC (executive regions). This indicates that in flow, the salience network strongly engages the ECN—aligning with the idea that the brain is in a task-focused mode, with salience detection (perhaps of ongoing feedback or performance) feeding into executive adjustments. At the same time, they found decreased functional coupling between the rAI and the ventral striatum (a key reward region) during flow. Interestingly, they did not find significant coupling changes between the rAI and the mPFC (DMN hub) during flow. Nor was there a change with the amygdala. The reduced insula-striatum connectivity in flow (relative to other states) might indicate that under the high salience of an engaging autotelic task, the salience network focuses on engaging control networks and less on tagging reward value—possibly because the task is intrinsically rewarding and does not require additional dopaminergic “boost” beyond a certain point. Another interpretation is that during flow, because one is already in an optimally motivated state, the salience network’s job is to sustain performance (*via* ECN) rather than keep checking the reward outcome. In any case, the pattern of increased insula–PFC connectivity fits the notion of networks synchronizing: the salience network recruits the ECN while presumably suppressing DMN interference. This is consistent with other work suggesting the insula helps disengage the DMN when attention is required.

Ulrich et al. (2022b) tested whether previously reported neural activation “signatures” of flow replicate in a fresh sample using the same boredom–flow–overload arithmetic paradigm. In 41 healthy male participants scanned with BOLD fMRI, they quantified replication using replication Bayes factors and found decisive replication evidence for the two hallmark quadratic patterns: an inverted U-shaped activation (higher during flow than boredom and overload) in regions including the DLPFC, anterior insula, and parietal cortex, and a U-shaped activation (lower during flow than boredom and overload) in regions including the mPFC, ventral striatum, amygdala, and cingulate cortex. They also showed strong replication evidence for the expected electrodermal activity pattern, supporting the robustness of the task manipulation, though subjective flow ratings could not be fully analyzed due to an item error.

In terms of interpretation for network-level accounts of flow, this replication strengthens the idea that flow reliably involves a rebalancing between task-positive control/salience systems (e.g., DLPFC and anterior insula showing the “flow peak”) and core default-mode/self-referential regions (e.g., mPFC showing the “flow dip”). Even without direct connectivity estimates, the replicated pattern is consistent with the broader claim that flow reflects a coordinated brain state in which attention/control-relevant regions are preferentially engaged while self-referential and affect-related regions are relatively downregulated during optimal engagement.

In summary, connectivity evidence from multiple angles suggests that flow involves a specific state of network integration. When in flow, individuals seem to achieve a brain state where task-positive networks (executive, attentional) are highly engaged and even working cooperatively with normally task-negative networks (default mode) or with the reward system, whereas in non-flow states these networks either disengage or interfere with each other. This likely provides the neural basis for why flow feels both effortless and highly focused: the brain’s networks are in sync, minimizing internal conflict between focus and spontaneity. The DMN is not entirely shut off; rather, it might contribute internally generated ideas or spontaneous associations (needed for creativity), but the ENC is simultaneously active to immediately harness those ideas toward the task goal. The result is an experience of fluid thinking where ideas come readily but also fit the task demands—exactly the balance needed for creative flow.

#### 3.2.4 Emotional and reward-related findings

Although not the primary focus of all studies, several findings provide data which inform how flow might regulate emotion. The amygdala is central to processing stress and fear; its downregulation in flow suggests a neurobiological correlate of the low anxiety and diminished sense of threat in this state. Participants in flow reported the experience as positive and not characterized by worry, which corresponds to this reduced amygdala activation. Ulrich et al. (2016) did not explicitly report the amygdala (aside from confirming the 2014 pattern). However, Ulrich et al. (2022a) did not find a significant connectivity change involving the amygdala—implying that the amygdala’s role may be more about overall arousal level than network coupling. At the same time, the replication study by Ulrich et al. (2022b) provides additional support that amygdala and ventral striatal involvement is a robust feature of the flow paradigm at the activation level, reporting decisive replication evidence for a U-shaped pattern in regions including the amygdala and ventral striatum (i.e., lower activation during flow relative to boredom and overload).

Another emotionally relevant aspect concerns how striatal and control-related circuits vary with engagement. In the naturalistic gaming paradigm of Huskey et al. (2018), the balanced difficulty condition, intended to approximate flow, was associated with the highest self-reported intrinsic reward/engagement and with PPI connectivity effects reported using the nucleus accumbens as the seed, indicating that striatal coupling with frontal control-related regions differs as a function of engagement state. However, the study design does not support strong, reward-specific mechanistic claims: connectivity results were seed-dependent (a significant DLPFC–accumbens connection was not reported when DLPFC served as the seed), and the inclusion of a secondary reaction-time task means basal ganglia findings may partly reflect dual-task control demands rather than reward processing per se. Interpreted conservatively, these results suggest an engagement-linked striatal–frontal interaction pattern that accompanies optimal challenge–skill matching, while leaving open whether the underlying driver is reward, cognitive control, or their interaction. In the low- and high-difficulty conditions, Huskey et al. also reported greater involvement of default mode regions, consistent with disengagement or task-irrelevant thought when challenge and skill are mismatched.

It is worth noting that none of the included studies explicitly measured “emotion regulation” abilities; however, the patterns observed (reduced amygdala, low self-referential processing, high intrinsic reward) are consistent with a brain state conducive to stable, positive mood. One study by de Sampaio Barros et al. (2018) incorporated a stress manipulation prior to the task and found that, even after a social stress test, those who achieved flow had certain physiological profiles (e.g., higher heart rate variability) indicative of efficient regulation of stress responses. Although not a primary outcome, this suggests that flow can occur in spite of prior stress and may even dampen its impact, as seen in the autonomic patterns associated with flow (higher parasympathetic activity correlating with better emotional control). In the context of creativity, flow’s emotional facet (enjoyment, intrinsic motivation) may create a mental environment where novel ideas are more freely generated and less likely to be hindered by fear of failure or criticism. This appears to resonate with Beaty et al. (2016) finding that global efficiency of the creative brain network correlated with divergent thinking ability—possibly those with more positive, less inhibited mindsets (common in flow) harness their brain networks more efficiently for creativity.

## 4 Discussion

### 4.1 Main findings

This systematic review synthesizes current neuroimaging evidence on how flow states modulate large-scale brain networks, particularly the DMN and ECN, and the implications for creativity and emotional regulation. Despite diverse tasks and methodologies, a credible story emerges: flow states correspond to a distinctive brain configuration characterized by both segregation and integration of neural networks. On the one hand, regions associated with self-focused, inward thinking (DMN) are consistently downregulated during flow, reducing internal distractions and negative emotions. On the other hand, task-focused networks (ECN and attentional circuits) are activated and, in some studies, functionally coupled with other networks (including DMN nodes and reward circuits) in ways that do not typically occur in ordinary states of consciousness. Importantly, the robustness of the core activation-based “flow effects” (boredom–flow–overload contrasts) is supported by a quantitative replication demonstrating decisive evidence for both major neural patterns, including regions spanning executive/salience-related cortex and DMN/limbic–striatal regions; however, that replication also notes a limitation in the subjective flow-experience index due to an item-implementation error, underscoring that neural signatures are not always paired with a fully intact self-report flow composite across all studies.

Building on the foundational work of Csikszentmihalyi et al. (2014), we find that flow emerges most robustly when an individual’s perceived challenge is balanced with their skill level, creating a state of optimal engagement and immersion. All included studies support some aspect of the hypothesis that flow involves optimized network dynamics. When challenge and skill are balanced, individuals appear to enter a state of effortless attention that is reflected in the brain by reduced activity in midline default mode regions (mPFC, PCC) and increased activity in control regions (DLPFC, frontal cortex) and often salience/reward regions (insular cortex, striatum, amygdala). This pattern aligns with theoretical models proposing that flow is a state of transient hypofrontality plus hyper-focus. However, our review nuances this view: rather than a blanket “hypofrontality” (shutdown of frontal executive function)—which was theorized by Dietrich (2004)—the evidence suggests a more selective attenuation of self-monitoring aspects of frontal function (mediated by mPFC and orbitofrontal areas) combined with preservation or even enhancement of task-related frontal function (DLPFC, IFG for cognitive control). In experts, we do see broad prefrontal deactivation during creative flow (as in jazz improvisers), which likely indicates that once a high level of skill is achieved, the brain can afford to “quiet down” even parts of the control network. But in non-experts or more analytic tasks, flow may still require significant frontal engagement to maintain concentration (e.g., the IFG activation in flow during math tasks). Thus, flow might lie at the sweet spot of frontal activation—enough to focus, not so much as to metacognitively worry.

The DMN silencing in flow dovetails with subjective reports of diminished self-awareness. Interestingly, similar DMN reductions occur in mindfulness meditation and certain altered states, which are also marked by loss of ego-centric processing. The unique aspect of flow, however, is that it occurs concurrently with high cognitive performance. In depression or rumination, DMN is overactive and coupled with emotional circuits leading to maladaptive self-focus; in flow, we see the opposite: DMN is tamped down, freeing the person to immerse in the task, which is generally a positive and productive state. This suggests flow could be a target state for interventions aiming to reduce maladaptive self-referential thought (e.g., in anxiety or depression)—essentially harnessing attention to break the cycle of rumination. The reviewed studies lend neurological credence to that idea, showing consistent DMN reductions and, in the replicated flow paradigm, robust involvement of limbic/striatal regions alongside midline DMN regions, even as generalizability remains limited by the repeated use of men-only samples in that paradigm.

### 4.2 Implications for creativity

One of the strongest themes in our findings is the support for the notion that flow is conducive to creativity, and this is underpinned by DMN–ECN connectivity. The studies by Beaty et al. (2016, 2018) provide convergent evidence that creative cognition is supported by coordinated coupling between default-mode and executive-control systems, consistent with the idea that creativity benefits when spontaneous idea generation is shaped in real time by goal-directed selection and constraint. In a sense, being in flow might mimic what highly creative brains do naturally—engage multiple neural systems in parallel. For instance, a writer “in the zone” might be tapping default mode processes (spontaneous imagination) and executive processes (choosing words, following narrative structure) simultaneously. Our review finds direct evidence of such parallel processing in the brain. The fact that greater DMN–ECN connectivity was associated with better divergent thinking suggests that training or techniques that encourage this integration (perhaps *via* fostering flow) could enhance creativity. Conversely, creative people might be more prone to experiencing flow because their brains more readily enter this integrated network state. This aligns with anecdotal accounts of artists and scientists seeking flow to do their best work. From a practical standpoint, environments that facilitate flow (clear goals, balanced challenge, minimal distractions) could be fertile grounds for creativity because they orchestrate the brain into this productive rhythm.

Another interesting point is the role of the salience network, particularly the right anterior insula, as studied by Ulrich et al. (2022a). The salience network may act as a mediator or switch that helps the brain navigate between exploratory (DMN-driven) and focused (ECN-driven) modes. In flow, the insula’s heightened connectivity with ECN and reduced connectivity with striatum might indicate a state where the salience network prioritizes task-related stimuli and performance feedback over internal or reward signals. This is adaptive: when in flow, people often report a loss of conscious concern even about the reward—they do the activity for its own sake (autotelic experience). The brain findings suggest that the usual tight coupling between doing well and feeling rewarded might loosen (the insula is less coupled with the reward center), so that attention remains on the activity, not on the “prize” or outcome. Paradoxically, by not focusing on reward, one might perform better and eventually achieve greater rewards—a phenomenon many athletes and creators describe. A replication study from the same group (Ulrich et al., 2022b) strengthens confidence that core flow-related network signatures are robust—reporting strong/decisive replication evidence for the characteristic quadratic activation patterns across key regions (including control/salience-related areas such as DLPFC/anterior insula and default/affective regions such as mPFC and amygdala). However, because this replication work did not assess creative performance outcomes, its relevance here is supportive (mechanistic plausibility and reliability of the flow signature) rather than direct evidence linking flow to creativity.

The creativity outcomes discussed here primarily reflect generative and fluent creative cognition/performance, including self-perceived creativity during artmaking (Cseh et al., 2015), divergent thinking (Beaty et al., 2016), and real-time musical improvisation under varying expertise (Rosen et al., 2024). Because later-stage creativity emphasizing extended planning, evaluation, and refinement is not directly tested in these paradigms, we avoid generalizing any “hypofrontality” account to all forms of creative behavior. Within the included creative-performance context, higher self-rated flow during improvisation was accompanied by reduced frontal activity, particularly among experts (Rosen et al., 2024). By contrast, in flow-induction paradigms assessed with hemodynamic methods, flow was accompanied by reduced activity in core default-mode hubs (e.g., mPFC/PCC) alongside engagement of task-positive control/attention regions (Ulrich et al., 2014; Ulrich et al., 2016), with complementary evidence for frontoparietal attentional engagement during flow from NIRS (de Sampaio Barros et al., 2018). Together, these patterns motivate a phase- and expertise-sensitive account of when reduced frontal activity may be beneficial vs. detrimental (Dietrich, 2004).

Evidence for reduced lateral PFC activity is most consistently discussed in highly practiced, time-pressured, performance-like contexts—for example, musical improvisation—where excessive top-down monitoring could disrupt well-learned sensorimotor and associative routines. In such cases, reduced engagement of dorsolateral prefrontal regions has been interpreted as a functional “letting go” of overt executive interference, potentially enabling fast retrieval and recombination of learned patterns during spontaneous creation.

At the same time, converging evidence indicates that creative quality can increase with stronger recruitment of prefrontal control systems, especially when tasks demand controlled semantic search, selection among competing candidates, and suppression of dominant but unoriginal responses. In an fMRI study explicitly contrasting idea generation demands, higher creative quality was associated with stronger activation in left prefrontal regions (including inferior/orbital frontal areas), consistent with a role for controlled retrieval and constraint implementation during divergent thinking (Benedek et al., 2014). Complementing this, resting-state work comparing high- vs. low-divergent-thinking individuals found that higher creative ability was associated with greater functional connectivity between inferior frontal control regions and default-mode hubs, as well as links involving dorsolateral PFC (Beaty et al., 2014), suggesting that effective creativity often reflects integration of spontaneous and control processes rather than a uniform reduction of executive involvement.

Taken together, we do not interpret the current evidence as supporting the view that all creative behavior is associated with flow and prefrontal hypoactivity. Instead, reduced lateral PFC engagement may be beneficial during fluent generation or performance in experts (minimizing self-monitoring costs), whereas sustained PFC engagement is likely beneficial, and sometimes necessary, during evaluation/refinement, rule maintenance, and long-term implementation of creative ideas.

### 4.3 Implications for emotional regulation

While none of the experiments directly tested emotion regulation (e.g., no one did a stress reappraisal task in flow vs. out of flow), the results allow us to cautiously infer some possible regulatory benefits. Beyond creativity, flow likely influences emotional regulation through partly overlapping network mechanisms, including shifts in self-referential processing, attentional engagement, and affective valuation. Flow is characterized by positive affect and an absence of negative, intrusive thoughts; in neural terms, this pattern is consistent with findings of reduced amygdala activity and reduced DMN self-related processing reported in flow-induction paradigms (Ulrich et al., 2014; Ulrich et al., 2016). This combination resembles the aim of many emotion-regulation strategies: decreasing negative emotion (amygdala-linked arousal) while shifting attention away from self-focused rumination (DMN-linked self-referential thought). Flow may achieve this “regulatory” profile naturally, as a side-effect of intense engagement, which is why it might function as a protective state against stress. Notably, this affective/reward-related component is further supported by the replication study (Ulrich et al., 2022b), which reported decisive replication evidence for flow-related quadratic activation patterns that included the amygdala and ventral striatum, alongside strong replication evidence for electrodermal activation as a psychophysiological readout of the flow manipulation.

At the same time, it is worth noticing that flow and emotional regulation engage overlapping but distinct neural circuits. Deliberate emotion regulation (like cognitive reappraisal) typically recruits higher prefrontal control. Adaptive regulation strategies such as reappraisal or mindfulness show greater engagement of the mPFC, together with the amygdala, suggesting top-down modulation that still involves emotional processing, whereas maladaptive regulation (e.g., expressive suppression) leans more on dorsal PFC activity with reduced amygdala activation (Barnett and Vasiu, 2024). In other words, reappraisal appears to use an mPFC “high road” that engages feelings while reframing them, whereas suppression appears to use a DLPFC “high road” to inhibit emotional responses.

These distinctions help contextualize the flow state: during flow/creative immersion, reduced DLPFC activity (especially in skilled performance) can be interpreted as less self-conscious evaluation, while mPFC and limbic circuits can still contribute affective information that is integrated into the task. One study in our review found that during tasks designed to induce flow, individuals who experienced greater flow reported higher heart rate variability, which is a marker of adaptive emotion regulation. This suggests that flow may involve net activation of the parasympathetic nervous system despite high levels of focus, supporting the idea that flow states are physiologically distinct from stress states and may promote emotional resilience (de Sampaio Barros et al., 2018). At the same time, reduced recruitment of frontal control systems is not universally adaptive: in other contexts (e.g., task-based fMRI meta-analyses in ADHD), reduced activation within frontoparietal/control circuitry has been linked to difficulties in attention and executive regulation (Cortese et al., 2012).

Even in the improvisation context, Limb and Braun (2008) observed that deactivation in the lateral orbitofrontal cortex may indicate a reduced engagement of functions supported by this region, including aspects of impulse control, highlighting a plausible boundary condition: reductions in control-related prefrontal systems may be helpful during certain generative phases but could be counterproductive when creative work requires sustained regulation, monitoring, and implementation.

Taken together, the current evidence is most consistent with a nuanced account in which flow can resemble a naturally occurring “regulatory mode” by quieting self-focused thought and reducing intrusive negative affect while maintaining strong task engagement. However, this does not imply that reduced prefrontal involvement is universally beneficial: depending on the demands of the situation, downshifts in control-related frontal systems may support effortless absorption and reduced self-monitoring, or they may compromise impulse control, sustained monitoring, and goal maintenance. We therefore treat any regulatory advantages of flow as likely to be both phase- and task-dependent, and as hypotheses that require direct testing with designs that manipulate emotional challenge and quantify regulation success during flow.

### 4.4 Clinical implications

Flow experiences may confer tangible physical health benefits. In a large longitudinal twin study (>9,000 people), individuals with higher flow proneness had significantly lower risk of stress-related disorders and even cardiovascular disease over time (Gaston et al., 2024). Specifically, those more inclined to experience flow showed a reduced incidence of depression and anxiety, and a ~ 4% lower risk of developing cardiovascular conditions in the follow-up, even after accounting for genetic factors and personality (Table 4).

**Table 4:** Summary of findings from Gaston et al. (2024).

Health diagnosis	Risk reduction with higher flow proneness	Risk reduction after controlling for neuroticism	Risk reduction in monozygotic twins (more flow vs. co-twin)
Depression	16% (CI [14, 18%])	6% (CI [3, 9%])	16% (CI [5, 26%])
Anxiety	16% (CI [13, 18%])	5% (CI [1, 8%])	13% (CI [1, 24%]) (non-significant after controlling for neuroticism)
Schizophrenia	15% (CI [4, 25%])	Not reported	Not reported
Bipolar disorder	12% (CI [6, 18%])	Not reported	Not reported
Stress-related disorders	9% (CI [9, 12%])	Not reported	Not reported
Cardiovascular disorders	4% (CI [1, 8%])	Not reported	Not reported

It is worth mentioning that flow states share properties with established mind–body practices (e.g., meditation or yoga) by concurrently engaging focused attention and physiological regulation. Recent work suggests that experiencing flow can lower stress and negative mood—for instance, flow proneness predicts reduced anxiety and depression (Asakawa, 2010)—and may co-activate parasympathetic systems (Peifer and Tan, 2021). In this light, clinicians can treat flow as a self-induced mind–body intervention: a positive, immersive state that can improve mental and physical wellbeing. Framing flow as a rehabilitative modality thus encourages therapists to intentionally induce it, much like other interventions that occupy the mind to reduce rumination and stress (Rankin et al., 2019).

Implementation within clinics could consider two slightly distinct protocols, depending on whether the practitioners are novice or experienced. Beginners should likely engage in longer, frequent practice of enjoyable creative or physical activities (for example, art, music, dance, or mild sports). The client should practice until a level of automaticity is reached, then gradually increase session length. Design can influence outcomes; it is typically helpful to emphasize clear goals and immediate feedback (e.g., learning to play a simple melody until mastery, then adding complexity) (Yoshida et al., 2018). For skilled individuals, clinicians can focus on goal-setting and adaptive challenges. Literature shows that flow is easiest to sustain when challenge and ability are closely matched; as skill grows, raising the bar is essential to prevent boredom (Ottiger et al., 2021). Clinicians can also use challenge-skill assessment (as in occupational therapy) to tailor task difficulty (Ottiger et al., 2021). Finally, clinicians can encourage clients to keep a “flow diary” to note conditions that trigger flow and reflect on their experiences. As clients practice, skills become more automatic—a shift from explicit (conscious) control to implicit competence (Dietrich, 2004). Over weeks, this means clients can perform tasks with more ease, further facilitating flow.

Integrating flow state-oriented activities alongside conventional medical treatment may also enhance therapeutic outcomes. By encouraging patients to engage in personalized, skill-based creative or physical practices that foster flow, clinicians could observe whether these experiences amplify the effects of standard treatment protocols. For example, flow-promoting programs may support better mood regulation, increase motivation, improve adherence to treatment, and reduce perceived symptom burden. Over time, clinicians might track whether patients who engage in flow-enhancing routines demonstrate greater improvements in clinical measures, such as pain, fatigue, or emotional resilience, than those receiving standard care alone. This integrative approach could offer a promising, low-cost adjunct to conventional protocols—particularly for individuals managing chronic conditions where self-efficacy and engagement play a critical role in healing.

### 4.5 Limitations and future directions

Several limitations were identified. Sample sizes were small in several studies, which means some results could be due to chance or might overestimate effect sizes. The tasks used to induce flow were also relatively short and artificial (except the music improvisation). Real-life flow (like in a long athletic competition or painting for hours) might engage additional processes and networks (for instance, motor networks, or deeper reward systems) that were not captured fully here. Also, individual differences were not the focus of most included studies—but in practice, people differ in how easily they experience flow (trait flow), and that might correlate with stable brain differences (like the PET finding of more dopamine receptors in flow-prone individuals). None of the included studies examined neurotransmitters directly except indirectly *via* blood flow or coupling changes; thus, the neurochemical aspect of flow (e.g., dopamine release) is a missing puzzle piece in this review; however, prior research suggests dopamine is involved in the rewarding aspect of flow (De Manzano et al., 2013).

Another limitation is that creativity in these studies was mostly measured as divergent thinking or musical improvisation. These are valid, but creativity is multifaceted. It would be useful to see if the flow-network relationship holds for other creative endeavors (writing, design, scientific problem-solving). The emotional side, similarly, was inferred, not measured: future studies could explicitly test if people in flow show better down-regulation of negative emotion (e.g., by introducing an emotional distractor during flow and seeing if the brain/emotion is less perturbed than when not in flow).

We note that replication efforts are emerging for the most commonly used experimental flow paradigm. In a fresh sample (*N* = 41), Ulrich et al. (2022b) explicitly quantified replication evidence using replication Bayes factors, reporting strong replication evidence for electrodermal activation and decisive replication evidence for the two core neural ‘flow effects’ (inverted U-shaped and U-shaped activation patterns). This partially mitigates concerns that earlier findings were purely chance-driven within this paradigm, while still leaving key generalizability gaps—most notably the continued reliance on men-only samples and the need for broader replication across diverse populations, tasks, and analytic pipelines.

This field is still in an early stage, and our review points to several future research directions. First, more integration of creativity measures into flow experiments would be valuable—for example, having participants complete a creative task in the scanner and measuring both their flow state and creative outputs to directly link network connectivity with creative performance. Second, longitudinal or training studies could test causality: if you train someone to achieve flow (e.g., through biofeedback or skill learning), do their DMN–ECN connectivity patterns change over time? And does that improve creativity or emotion regulation in daily life? Third, exploring individual differences (e.g., comparing high-flow-proneness individuals to low-flow-proneness) could reveal trait neural markers—maybe some people’s brains are wired to more easily disengage DMN and synchronize networks, which could explain why they experience flow often. That could have implications for occupational or educational settings (tailoring activities to individuals). Fourth, investigating flow in populations with attention or mood disorders might be insightful—can flow be induced in those with depression, and would it temporarily normalize their DMN hyperactivity? Preliminary evidence from mindfulness research would suggest, possibly, yes. Neurofeedback to mimic flow-like brain states might even be an intervention avenue.

From a network neuroscience perspective, flow is a compelling example of how the brain’s large-scale networks can reconfigure in a highly advantageous way. Modern techniques such as dynamic functional connectivity and graph-theory metrics could be employed in future to quantify the “network flexibility” or efficiency during flow. For instance, is the global efficiency of brain connectivity maximal during flow? Are there certain hub nodes that become more central (e.g., insula, dorsolateral PFC) when we enter flow? Such analyses could deepen our understanding of the flow state.

It is worth bridging the findings on DMN suppression in flow with the older concept of the “transient hypofrontality hypothesis.” Our review indicates that it is not a full shutdown of the frontal cortex, but a selective one. This nuance was actually foreshadowed by those who suggested flow might involve a transient reorganization rather than uniform deactivation. Our included results confirm a specific pattern: the lateral PFC (for task) stays active or even increases, while the medial PFC (self-focused) decreases. This pattern effectively reconciles the apparent contradiction between needing focus (frontal function) and losing self-awareness (frontal function)—it is two different parts of the frontal lobe doing different things.

Finally, flow can be considered a valuable tool in therapeutic interventions. Interventions that specifically stimulate flow states could optimize emotional regulation by promoting deep absorption, reducing negative self-focus, and increasing intrinsic motivation. Unlike cognitive approaches that rely on explicit reappraisal (which engages mPFC-amygdala circuits), flow can be used to shift attention away from stressors through task immersion and neurochemical reward reinforcement. Given its links to autonomic regulation and stress resilience, structured flow-based activities—such as guided artistic creation, movement-based improvisation, or interactive gaming—could be integrated into therapy to complement existing creative, cognitive, and mindfulness-based interventions. Future research should explore whether fostering flow states in integrative therapies adds value in terms of improving psychological outcomes (e.g., reduced rumination and anxiety) and physiological markers of wellbeing, such as heart rate variability, cortisol levels, and inflammatory markers.

## 5 Conclusion

This systematic review highlights the role of functional connectivity between the DMN and ECN in facilitating flow states and supporting their contributions to creativity and emotional regulation. Flow is characterized by reduced self-referential processing in the DMN and increased cognitive control *via* the ECN, allowing for heightened focus and optimal task engagement. Studies show that flow enhances creative performance by integrating spontaneous ideation with goal-directed action, while also promoting emotional stability through the downregulation of stress-related brain regions and increased intrinsic motivation. This network synchronization allows individuals to sustain deep involvement in tasks with minimal cognitive interference, creating an ideal environment for both productivity and psychological resilience.

Beyond its implications for creativity, flow appears to be a natural mechanism for emotional regulation, dampening excessive self-monitoring and negative affect. The neurophysiological markers of flow suggest that it facilitates a shift away from maladaptive cognitive patterns, making it a potentially valuable framework for clinical applications, including in anxiety, depression, attention-related disorders, schizophrenia, stress-related disorders, bipolar disorders, and cardiovascular disorders. Additionally, findings indicate that individuals who more readily experience flow may possess greater neural efficiency in integrating task-relevant brain networks, a factor that could inform strategies for optimizing learning and performance in educational and professional settings.

Future research should further investigate the causal mechanisms underlying DMN-ECN connectivity in flow states, explore the roles of neurotransmitters such as dopamine and serotonin in sustaining this state, examine individual differences in flow susceptibility, and further explore the psychological and physical benefits of integrative interventions incorporating flow. Understanding how flow modulates brain activity will not only advance theoretical models of cognitive function but also inform interventions aimed at enhancing performance, wellbeing, and creative potential across various domains.

## Data Availability

The original contributions presented in the study are included in the article/supplementary material, and further inquiries can be directed to the corresponding authors.
